# Cuproptosis in lung cancer: a nexus of ncRNA regulation, epigenetics, and tumor microenvironment Remodeling

**DOI:** 10.1007/s10238-025-01903-9

**Published:** 2025-12-03

**Authors:** Mohamed S. Imam, Randa M. Abdel-Sattar, Nasser M. Aldekhail, Norah Khalid Abdullah Humaish, Shoug Abdulaziz Gary Gary, Mansour Abdulrahman Mansour Alkhulaifi, Misk Abdullah Mohammed Alqahtani, Malak Lafi Zaid Aldajani, Hasna Mohammed Jarallah Altuwayhir, Wasan Izzualdien Abdulrahman Alnaim, Aldanah Hmoud Alotaibi, Reem Jazzaa S. Alotaibi, Ahmed M. Mayla, Yasser Mabrouk Bakr

**Affiliations:** 1 Department of Clinical Pharmacy, National Cancer Institute, Cairo University, Fom El Khalig Square, Kasr Al-Aini Street, Cairo, 11796 Egypt; 2 Department of Pharmaceutics, College of Pharmacy, Shaqra University, 11961 Shaqra, Saudi Arabia; 3 Department of Pharmacy, College of Pharmacy, Nursing and Medical Sciences, Riyadh Elm University, Riyadh, Saudi Arabia; 4 College of Pharmacy, Taif University, 21944 Taif, Saudi Arabia; 5 College of Public Health and Health Informatics, University of Ha’il, 55476 Ha’il, Saudi Arabia; 6 College of Clinical Pharmacy, King Faisal University, Al-Ahsa, Eastern Province Saudi Arabia; 7 College of Pharmacy, Shaqra University, 11961 Shaqra, Saudi Arabia; 8 Virology and Immunology Unit, Cancer Biology Department, National Cancer Institute (NCI), Cairo University, Cairo, 11796 Egypt; 9 National Cancer Institute, Cairo University, Fom El Khalig Square, Kasr Al-Aini Street, Cairo, Egypt

**Keywords:** Lung cancer, Cuproptosis, Non-coding RNAs, Epigenetics, Tumor microenvironment, Biomarkers, Targeted therapy

## Abstract

Lung cancer remains a leading cause of cancer-related mortality worldwide. Cuproptosis, a new form of programmed cell death, is emerging as a key regulator in tumor progression. In this review, we talk about the interplay between cuproptosis, non-coding RNAs (ncRNAs), and epigenetic modifications in lung cancer. We performed an extensive review of recent literature to explore the function of ncRNAs in the regulation of cuproptosis, their effects on tumor microenvironment remodeling, immune response regulation, and drug sensitivity. ncRNAs were found to modulate cuproptosis by influencing copper metabolism, apoptosis, and oxidative stress response. Specific ncRNA signatures possess prognostic biomarker and therapeutic target potential in lung cancer. In addition, ncRNA-mediated epigenetic regulation has significant influence on deciding lung cancer formation and treatment outcome. The integration of non-coding RNAs related to cuproptosis into therapies offers great promise for the improvement of lung cancer prognosis. Further studies are needed to validate these findings and promote their implementation in clinical practice.

## Introduction

Lung cancer has a significant global health burden, with high morbidity and fatality rates. It is typically divided into two types: non-small cell lung cancer (NSCLC) and small cell lung cancer (SCLC), with NSCLC accounting for around 85% of all lung cancer cases [1, 2]. The two most prevalent NSCLC subtypes are lung adenocarcinoma (LUAD) and lung squamous cell carcinoma (LUSC) [1]. LUAD, the most diagnosed histopathological subtype, represents 40–55% of NSCLC cases. It originates from small airway epithelial cells and type II alveolar cells, and its incidence is increasing among smokers, former smokers, and non-smokers alike [3]. LUAD is a markedly diverse disorder characterized by intricate etiology, which complicates treatment efforts [3]. LUSC, on the other hand, constitutes 20–30% of NSCLC cases and is regarded as one of the most difficult tumors to manage [4, 5]. Despite progress in treatment options, long-term survival rates remain poor [2]. The five-year survival rate for LUAD is approximately 18%, while for NSCLC overall, it is around 15.9% [1]. Conventional treatments such as chemotherapy, surgery, and radiation therapy face limitations, including drug resistance and adverse side effects [2]. Furthermore, tumor heterogeneity and the absence of effective targeted therapies add to the challenges of managing this disease [1].

Cuproptosis is a newly identified paradigm of controlled cell death, separate from other mechanisms such as apoptosis, necrosis, autophagy, pyroptosis, senescence, ferroptosis and mitotic catastrophe [6, 7]. It is a copper-dependent process driven by the accumulation of copper ions (Cu^2^⁺) intracellularly [8]. The hazardous buildup of copper initiates this machinery, which interacts directly with lipoylated proteins involved in the tricarboxylic acid (TCA) cycle within the mitochondria [2]. Such binding gives rise to combination of lipoylated proteins and leads to a decline in iron–sulfur cluster proteins [9, 10]. The disruption of the TCA cycle results in mitochondrial dysfunction and reactive oxygen species (ROS) overload, triggering oxidative damage and ultimately cell death [11]. Cuproptosis is characterized by proteotoxic stress and does not need caspase activation, nor does it display the morphological traits associated with apoptosis [12].

In cancer biology, dysregulated Cu metabolism in tumor cells often results in elevated Cu levels [13], making them more susceptible to cuproptosis [14]. Moreover, Cu is a critical trace metal required for various normal cellular activities, including oxidative stress response, electron transfer, and enzyme functions [15]. However, it supports tumor growth, metastasis, and angiogenesis [10]. Cuproptosis can selectively target malignant cells, potentially reducing normal tissue injury [7, 8].. Considering this, scientists are exploring novel therapeutic approaches to leverage cuproptosis [16], Efforts include developing drugs and treatments that induce cuproptosis by modulating Cu metabolism [8], In addition, Cu chelators are being investigated for their ability to lower intracellular Cu levels [17, 18]. Additionally, Cu complexes are being targeted for their potential in cancer therapy [10]. Cuproptosis is linked to various tumor characteristics, such as tumor mutation burden (TMB) and the tumor immune microenvironment (TIME) [19, 20]. Numerous investigations indicated that cuproptosis plays a substantial part in a variety of tumors [21], embracing breast cancer [21] lung cancer [20, 22], hepatocellular carcinoma [23, 24], colorectal cancer (CRC) and prostate cancer [23], and pancreatic cancer [25].

The Long non-coding RNAs (lncRNAs) are revealed to be significant participants in cancer, particularly in LUAD, by influencing various cellular processes and acting as much as possible biomarkers and therapeutic targets [3]. Cuproptosis, a recently identified type of regulated cell death that relies on Cu levels, is an important research focus, with specific lncRNAs influencing regulatory functions [5]. Multiple studies have identified prognostic signatures based on cuproptosis-related lncRNAs (CRlncRNAs) for LUAD, demonstrating their power for patient survival and therapeutic response prediction [14, 26, 27]. These signatures often utilize machine learning techniques such as Least Absolute Shrinkage and Selection Operator (LASSO) and Cox regressions to pinpoint key lncRNAs [1]. Thus, lncRNA signatures can predict overall survival (OS), and other survival endpoints such as, progression-free survival (PFS) and disease-specific survival (DSS), often outperforming traditional clinical factors [28]. For example, a 13-lncRNA signature was found to be a reliable predictor of LUAD prognosis, while another study identified a 16-lncRNA signature linked to patient survival and treatment efficacy [27, 29]. Specific lncRNAs such as PVT1, LINC00853, LINC00592, ZNF571-AS1, and SEPSECS-AS1 have been highlighted for their roles in cancer, with their expression levels varying across cancer types and individuals [2, 30]. Furthermore, CRlncRNAs are related to the TIME, consequently they can expect response to immunotherapy [5]. Some studies indicate that individuals with low-risk ratings based on these markers may benefit from immunotherapy [5]. Additionally, these signatures are linked to TMB as well as the sensitivity of the drugs, making them beneficial for tailored therapeutic schemes [2, 31]. For example, a study identified potential drug candidates for patients with high-risk scores, including epothilone-b and gemcitabine [32]. Nomograms incorporating these lncRNA signatures with clinical parameters like age, stage, and gender can provide more accurate survival predictions for clinical use [1]. These lncRNAs may be able to prognosticate the effectiveness of radiotherapy in NSCLC cases [33]. Overall, the investigation of lncRNAs, especially those linked to cuproptosis, provides valuable insights regarding prognosis, diagnosis, and treatment of LUAD and other cancers [14, 34].

**Fig. 1:**
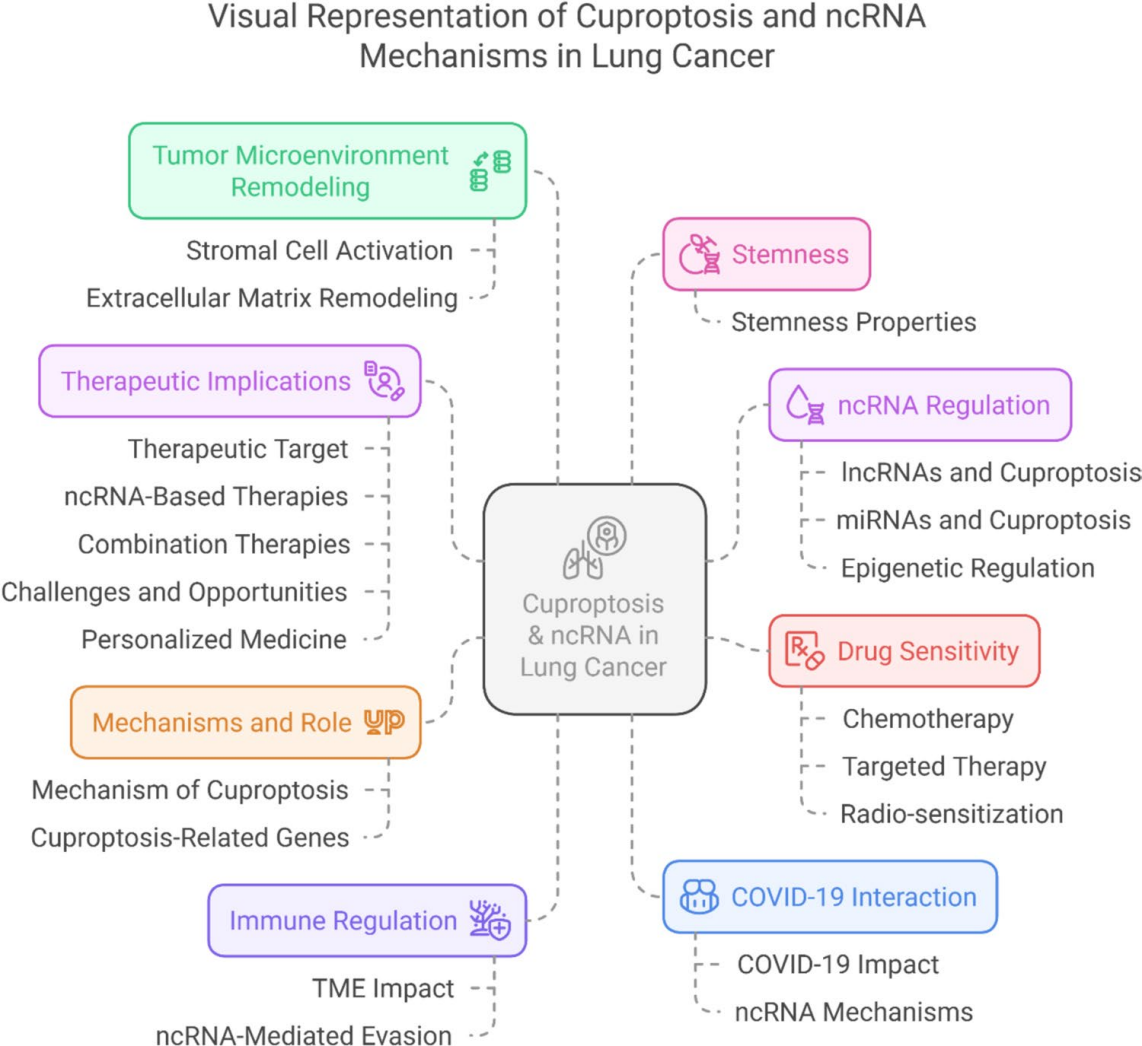
Schematic representation of cuproptosis and ncRNA mechanisms in lung cancer

Our objective is to highlight the interplay linking cuproptosis and ncRNA-mediated mechanisms in lung cancer, focusing on prognosis, immune regulation, drug sensitivity, and therapeutic potential as presented in Fig. 1.

## Cuproptosis: role and mechanisms in lung cancer

**Fig. 2:**
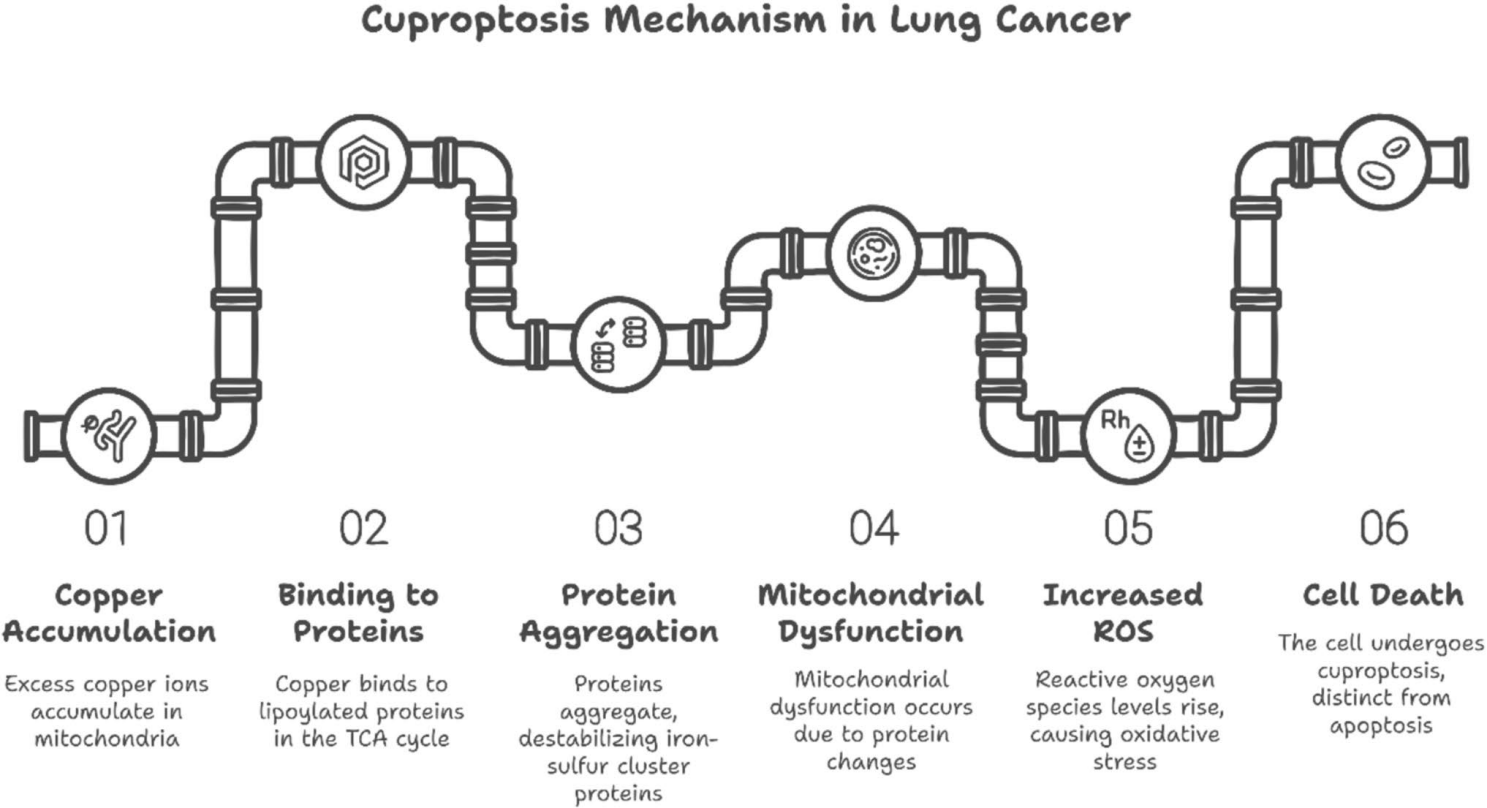
Illustrates the mechanism of cuproptosis, highlighting the role of copper accumulation and mitochondrial dysfunction

Cuproptosis is a form of cell death caused by the buildup of copper ions in mitochondria [11, 29, 35]. This process starts when excess Cu directly interacts with lipoylated proteins through the TCA cycle within the mitochondria, producing protein aggregates in addition to iron–sulfur cluster proteins deterioration [9]. This disruption makes a mitochondrial dysfunction, a free radical overrun, and subsequent redox damage, ultimately causing cell death [36]. Unlike apoptosis, it does not rely on caspase activation and lacks the morphological characteristics associated with apoptosis [35]. In lung cancer and other cancers, dysregulated Cu metabolism often leads to increased intracellular Cu levels, rendering tumor cells more vulnerable to cuproptosis [37, 38]. This dysregulation contributes to tumor progression, metastasis, and therapy resistance [10]. Specific cancers, such as lung adenocarcinoma (LUAD), exhibit high intracellular copper concentrations [39, 40]., and copper is known to be involved in promoting angiogenesis, which is condemnatory for neoplasm growth and metastasis [12]. Cuproptosis-related genes, including copper transporters such as CTR1, ATP7B, and ATP7A, are crucial for maintaining intracellular copper levels [41, 42]. Dysregulation of these transporters can lead to Cu buildup and subsequent mitochondrial dysfunction, triggering cuproptosis [43, 44]. Other genes involved in cuproptosis are related to the mitochondrial respiratory chain, the TCA cycle, and the ROS production, further influencing cellular responses to Cu stress [45, 46]. The expression of genes like NFE2L2, CDKN2A, DLAT, LIAS, LIPT1, and LIPT2 has been associated with cuproptosis and lung cancer [5, 47]. Therefore, cuproptosis, with its distinct mechanisms, offers a potential therapeutic avenue for selectively targeting cancer cells by leveraging their dysregulated copper metabolism [48–50], where this mechanism in shown in Fig. 2.

## ncRNA regulation of cuproptosis in lung cancer

lncRNAs and cuproptosis: The lncRNAs are pivotal in modulating cuproptosis in lung cancer by regulating the expression of genes and pathways involved in this process [3]. They influence the activity of Cu transporters, including ATP7A/B and CTR1, which are necessary for Cu homeostasis in the cell [42, 51]. Dysregulation of these transporters by lncRNAs can lead to Cu accumulation, triggering mitochondrial dysfunction and cuproptosis [41]. Additionally, lncRNAs can modulate genes involved in mitochondrial respiration and ROS production, thus regulating mitochondrial reaction to Cu-induced stress [42]. Specific lncRNAs, like LINC01232, have been shown to upregulate genes such as RAB22A through interactions with microRNAs [9]. Research has identified various lncRNA signatures, including cuproptosis-related, for lung cancer prognosis; for instance, one study highlighted 13 such lncRNAs [27]. Among these, lncRNAs like OGFRP1 and PTPRG-AS1 are involved in NSCLC progression [22, 52, 53]. PTPRG-AS1, for instance, promotes LUAD proliferation and decreases radiosensitivity in NSCLC [53]. Another study identified 1138 CRlncRNAs, with a subset of 8 lncRNAs used to develop a prognostic signature [21]. Further research has linked lncRNAs such as AC002467.1, LINC01740, and LINC02345 to cuproptosis in LUSC [5].

miRNAs and cuproptosis: MicroRNAs (miRNAs) modulate cuproptosis through competing endogenous RNA (ceRNA) networks, where lncRNAs can serve as miRNAs dockings [54–56]. For example, studies have shown that lncRNAs like miR-424-5p and AC144450.1 control the expression of CBX2 in lung cancer [57]. Knockdown of AC144450.1 leads to decreased CBX2 and increased miR-424-5p expression, indicating that the lncRNA acts as a ceRNA for the miRNA [57]. This mechanism is crucial in the development of neoplasms, where lncRNAs can operate as endogenous sponges influencing mRNA expression by sequestering miRNAs [57–59]. Dysregulation of miRNAs, like miR-7, which is aberrantly expressed in bronchogenic carcinoma, has been shown to influence mRNA expression of Raf1 along with EGFR [57].

Epigenetic regulation and cuproptosis: Epigenetic modifications play a vital role in controlling cuproptosis along with shaping cancer progression because they modulate ncRNA production and function, as well as cuproptosis-related genes. Among these modifications, m6A methylation, a prevalent RNA modification, serves as a key epigenetic mechanism that stabilizes and functionalizes ncRNAs. For instance, the m6A methylation level of lncRNA-XIST can impact its expression, therefore affecting the CRC proliferation and metastasis [60]. Furthermore, YTHDF3 has been shown to promote the degradation of m6A-modified lncRNAs, accelerating tumor development. These findings highlight the significant contribution of m6A-related lncRNAs to tumorigenesis and cancer progression [60].

With respect to bronchogenic carcinoma, m6A modifications on lncRNAs can impact their function in cuproptosis pathways, which can be challenging to study [60]. One study found that m6A-related lncRNAs in lung LUAD are associated with pathways like cell cycle, oocyte meiosis, and ubiquitin-mediated proteolysis. This suggests that m6A-modified lncRNAs can influence LUAD progression and overall survival (OS) [60].

Furthermore, m6A methylation-related genes show differential expression in bronchogenic carcinoma cases with high/low-risk scores, according to CRlncRNAs signatures [21]. Specifically, expression levels of METTL3, RBM15, YTHDC1, YTHDC2, and METTL14 can differ significantly between these groups, indicating a link between m6A methylation and CRlncRNA function [21].

Moreover, m6A RNA methylation modulators may serve as biochemical markers for LUAD prognosis, suggesting that the dysregulation of these regulators might aid in tumor development through altering the expression of lncRNAs and other RNAs involved in cuproptosis [20].

Epigenetic modifications can affect the transcription of cuproptosis-associated genes themselves. For instance, in human pan-cancer analyses, NFE2L2 has been recognized to be associated with the DNA methyltransferase expression [6]. This highlights the complex interplay between epigenetic modifications, ncRNAs, and cuproptosis-related genes, all of which can influence tumor progression [6].

ncRNAs modulate cuproptosis via LLPS regulation: The regulatory landscape of cuproptosis extends beyond lncRNAs and miRNAs to include other ncRNA species, most notably circular RNAs (circRNAs). These stable, covalently closed RNA molecules can act as efficient miRNA sponges, protein decoys, and even templates for translation, thereby adding another layer of complexity to the post-transcriptional control of cuproptosis-related genes [63, 71].

Liquid–liquid phase separation is a fundamental physicochemical process by which living cells form membrane-less, liquid-like biomolecular condensates [61–64]. These condensates compartmentalize specific biomolecules, such as proteins and nucleic acids, to facilitate various cellular activities, including the regulation of transcription, cellular stress responses, signal transduction, and maintenance of genome stability [61, 63, 64]. LLPS is driven by weak, multivalent interactions between biomolecules and is influenced by environmental conditions such as concentration, temperature, salt, and pH [61, 65]. Aberrant LLPS has been linked to various diseases, including neurodegenerative conditions and cancer [61, 66].

LncRNAs are highlighted as pivotal and direct regulators in cuproptosis-mediated cancer, primarily by acting as anchors or scaffolds for biomolecular condensates [62]. They intricately control gene expression at epigenetic, transcriptional, and post-transcriptional levels, impacting crucial processes like copper metabolism, mitochondrial stress responses, and apoptotic signaling, which are all integral to cuproptosis [3]. Examples include H19, which functions as a competitive endogenous RNA (ceRNA) by sponging various miRNAs (e.g., miR-29b-3p, miR-19b-3p, miR-675, miR-130a-3p, and miR-491-5p) to regulate gene expression and programmed cell death (PCDs) such as apoptosis and ferroptosis [67, 68], and also potentially modulates the PI3K/AKT pathway [67].

NEAT1 forms paraspeckle nuclear bodies involved in DNA damage response and p53 attenuation [69, 70], while MALAT1 can affect cuproptosis by modifying mitochondrial activity and oxidative stress responses [3, 71]. SNHG9 facilitates the phase separation of LATS1 to promote oncogenic YAP signaling [61, 72, 73], and NORAD sequesters Pumilio proteins in phase-separated bodies to maintain genome stability [64]. Other lncRNAs like HSATIII, TERRA, eRNAs, MajSAT, MELTF-AS1, LINP1, and 7SK are also implicated in LLPS processes related to DNA repair, transcription activation, and stress responses [62, 72, 74–76].

Beyond lncRNAs, microRNAs (miRNAs) are recognized for regulating phase separation in various LLPS structures [77]. The sources identify a "miRNA-mRNA network" responsible for the ectopic expression of cuproptosis genes [71]. However, the direct explicit link between miRNA-mediated LLPS and the specific cuproptosis mechanism is often indirect, such as through lncRNAs sponging miRNAs [67].

Circular RNAs (circRNAs) are also indicated to regulate phase separation in different LLPS structures [77]. Specific examples in hepatocellular carcinoma (HCC) include circVAMP3 promoting the phase separation of CAPRIN1, inhibiting HCC proliferation and metastasis [63, 71], and circRNA-YBX1 facilitating cytoskeletal remodeling via LLPS to attenuate liver cancer metastasis [63, 71]. While these show circRNA involvement in LLPS and cancer progression, the sources do not explicitly connect circRNA-mediated LLPS directly to the cuproptosis mechanism itself [63].

Additionally, ribosomal RNAs (rRNAs) and transfer RNAs (tRNAs) play important roles as components of various condensates formed by LLPS, such as stress granules and P-bodies [61, 78, 79]. Messenger RNAs (mRNAs), particularly those with N6-methyladenosine (m6A) modifications, can interact with binding motifs and phase-separate into compartmentalized condensates, including stress granules and P-bodies, to alter mRNA translation and degradation [61, 65, 80, 81]. This process is relevant to tumor development [61]. However, for these non-lncRNA types, while their involvement in LLPS and relevance to general cellular functions and cancer is acknowledged, the provided sources do not contain explicit statements directly linking the LLPS specifically mediated by these other RNA types to the direct modulation of the cuproptosis mechanism in the same detailed manner as described for lncRNAs.

Cuproptosis controlled by lncRNAs through cell cycle dynamics: Non-coding RNAs, particularly long non-coding RNAs (lncRNAs), have been found to significantly modulate cuproptosis through their influence on cell cycle regulation and cellular proliferation, especially in various cancer types [82]. Research highlights that lncRNAs are pivotal regulators in cellular processes, including cancer progression and stress response, and their involvement can trigger cell-specific responses to copper stress, thereby presenting novel opportunities for therapeutic targeting [3].

A prominent example of this modulation is seen with SNHG9 in prostate cancer (PCa) [83]. Functional experiments conducted on PCa cells (PC-3 and DU145), where SNHG9 is highly expressed, demonstrated that knocking down SNHG9 led to a significant increase in the proportion of cells in the G1 phase and a corresponding decrease in cells in the S and G2 phases [83]. This indicates that SNHG9 normally promotes DNA synthesis and cell cycle progression in PCa cells [83]. The inhibition of DNA synthesis and cell cycle progression resulting from SNHG9 knockdown was associated with increased apoptosis and the upregulation of key cuproptosis-related messenger RNAs (mRNAs), specifically FDX1 and DLST [83]. These findings collectively suggest that SNHG9 plays a critical role in PCa progression by activating cell proliferation, migration, and invasion, and its modulation directly impacts the cell cycle in a manner that influences susceptibility to cuproptosis [83]. Elevated SNHG9 expression is also correlated with poor prognosis in PCa [83].

Beyond SNHG9, other lncRNAs have also been implicated in cell cycle control and proliferation, linking them to cuproptosis:

- MALAT1 is known for its involvement in cell cycle control and metastasis [84–87]. Its effect on cuproptosis varies, potentially either protecting mitochondrial membranes or promoting copper-induced cell death by upregulating genes involved in mitochondrial reactive oxygen species (ROS) generation, depending on the specific cancer cell line [84–87]. In liver cancer, MALAT1 has been linked to tumor development partly through cell cycle modulation [87].
- H19 can modulate the PI3K/AKT signaling pathway, a pathway crucial for cell division and survival. By sponging miR-29a, H19 upregulates PI3K expression, which may counteract cuproptosis by promoting cell survival and proliferation in certain cancer types [3].
- PVT1 is associated with the regulation of the p53 pathway, which is essential for controlling the cell cycle and programmed cell death. Overexpression of PVT1 may stabilize mutant p53, allowing cancer cells to survive copper-induced stress [88–91].
- LINC00853 has been studied for its role in glycolysis and cell proliferation in pancreatic adenocarcinoma [87].
- NIFK-AS1, identified as a cancer-suppressing lncRNA, has been shown to arrest the proliferation and migration of tumor cells in the context of lung cancer [3].

These intricate relationships highlight that lncRNAs influence cancer cell survival and resistance to cell death by regulating fundamental hallmarks of cancer, including cell cycle progression and proliferation, which, in turn, impacts their susceptibility to cuproptosis. Understanding these mechanisms provides insights into how targeting cuproptosis-related lncRNAs could lead to novel therapeutic strategies to improve cancer treatment outcomes.

LncRNA modulation of cuproptosis via copper homeostasis and mitochondrial function in cancer: While long non-coding RNAs (lncRNAs) are recognized for their pivotal role in regulating cuproptosis pathways, the provided sources do not explicitly detail a mechanism where lncRNAs modulate cuproptosis directly by DNA damage response. Cuproptosis is a distinct form of programmed cell death triggered by the toxic accumulation of copper within cells, which disrupts mitochondrial function and leads to an overproduction of reactive oxygen species (ROS) [11, 46, 92]. Notably, cuproptosis is not dependent on caspase activation or shared morphological features of apoptosis [35, 36, 93–95].

However, the sources do indicate that copper can induce necrotic apoptosis via ROS-dependent DNA damage [96], and that the raised ROS per se damages cellular content and activates further cell stress/damage response, accelerating apoptosis in a feed-forward manner [35, 36, 93–95].

This highlights a link between copper, ROS, and DNA damage leading to apoptosis, but it is not presented as the mechanism by which lncRNAs directly modulate cuproptosis. Instead, lncRNAs play a crucial role in regulating cuproptosis-associated pathways by influencing copper metabolism and the expression of copper transporters like CTR1 and ATP7A/B [41, 42, 51], as well as by regulating genes related to mitochondrial respiration and the generation of ROS, which helps to fine-tune the mitochondrial responses to copper stress [41, 42, 51]. Furthermore, lncRNAs, such as PVT, have been linked to the regulation of the p53 pathway [3].

The tumor suppressor protein p53 is essential for controlling the cell cycle and programmed cell death in response to cellular stress [3]. Overexpression of PVT1 may stabilize mutant p53, potentially allowing cells to survive copper stress and influencing the ratio of apoptosis to cuproptosis [3]. Although p53 is a key player in DNA damage response, the sources frame this lncRNA-p53 interaction as an influence on cell survival and the balance between apoptosis and cuproptosis under copper stress, rather than explicitly as lncRNAs modulating cuproptosis through a DNA damage response itself.

The extensive regulation of cuproptosis by ncRNAs and epigenetics does not occur in a vacuum. These molecular changes profoundly influence the cellular dialog within the tumor microenvironment, particularly the immune response. As we will discuss in the next section, ncRNA-mediated control of cuproptosis directly shapes anti-tumor immunity and contributes to immune evasion.

## Cuproptosis and immune regulation in lung cancer

### Impact on tumor immune microenvironment (TME)

In lung cancer, cuproptosis is pivotal for shaping TIME through the gene regulation of immune checkpoints as well as the modulation of tumor infiltrating leukocytes [26]. In addition, research has demonstrated the ability of CRlncRNAs to deduce the LUAD immune landscape [22, 97]. For instance, high-risk cohorts identified by CRlncRNA signatures often display elevated stromal scores, reduced estimate scores, as well as diminished immune scores, indicating a less active immune environment [98–101]. In contrast, low-risk groups tend to exhibit greater immune cell infiltration and enhanced immune-related function scores [102]. This distinction is crucial, as it highlights the capacity of CRlncRNAs to influence immune cell activity within TME. Notably, specific CRlncRNAs correlated to key leukocytes’ infiltration, like macrophages, regulatory T lymphocytes (Tregs), and cytotoxic T lymphocytes (CTLs), thereby playing vital immuno-oncological roles [31]. In some research, a high-risk group characterized by CRlncRNAs had HLA-related pathways and significant upregulation of Type II interferon (IFN) Response [31].

### ncRNA-mediated immune evasion

The ncRNAs, exclusively lncRNAs, participate substantially in facilitating immune evasion in lung cancer [66–68]. Research suggests that lncRNAs can regulate the expression of immune checkpoints inhibitors like PD-L1 [103, 104]. For instance, elevated intra-tumoral Cu levels are contributing to cancer immune escape by enhancing the expression of PD-L1 [12, 105–107]. Certain lncRNAs may suppress immune responses by targeting PD-L1 or other immune check points, allowing tumor to evade immune detection and treatment. Additionally, some lncRNAs promote immune escape by modulating specific genes, such as GATA3, further aiding the survival and progression of cancer [2].

The link between cuproptosis and immunity is not monolithic. The data suggest a dual role: sufficient cuproptosis may release antigens and stimulate immunity, while chronic, sub-lethal copper stress may select for immunosuppressive phenotypes. This likely depends on the spatiotemporal dynamics of copper accumulation and the specific ncRNAs expressed, a crucial area for future spatial transcriptomic studies.

## Cuproptosis and COVID-19 interaction in lung cancer

The interplay between cuproptosis, ncRNAs, and immunity, as detailed in previous sections, becomes critically relevant in the context of external stressors like viral infection. The SARS-CoV-2 virus (COVID-19) provides a poignant clinical example of how an external trigger can hijack these pathways, exacerbating cuproptosis-related pathways in bronchogenic carcinoma [31]. Specifically, the virus appears to modulate the very cuproptosis-related ncRNA signatures we have described, leading to a dysregulated immune response and poorer outcomes.

### Impact of COVID-19 on cuproptosis

Research has revealed that COVID-19 cases of lung cancer experience much more bad prognosis [31]. This is partly due to the fact that COVID-19 infection can lead to the upregulation of cuproptosis-associated genes [31]. For instance, research has demonstrated that patients with both NSCLC and COVID-19 had higher levels of DLD, DLAT, NFE2L2, PDHA1, and GLS compared to those without COVID-19 infection. This upregulation suggests that COVID-19 infection exacerbates cuproptosis activity in lung cancer cells [31].

A 2024 study conducted by Li et al*.* supports these findings, revealing that COVID-19 infection independently contributes to worse PFS in patients with NSCLC. The research team identified twelve long non-coding RNAs (lncRNAs) that regulate cuproptosis using RNA-seq data from patient tissue samples, and they found that a high-risk group based on these lncRNAs had lower PFS rates and overall survival compared to the low-risk one. This implies that CRlncRNAs may be implicated in the poorer outcomes observed in NSCLC patients infected with COVID-19 [31].

### ncRNA-mediated mechanisms

Specific ncRNAs, especially lncRNAs, have been identified as key players in this interaction. The identified lncRNAs can be used to create risk scores that distinguish between patients at low risk and those at high risk. For example, lncRNA MAMDC2-AS1 is an important CRlncRNA in NSCLC, especially in the case of COVID-19 infection and has a significant prognostic value [31]. The expression of these lncRNAs may be altered due to COVID-19 infection, leading to triggering of cuproptosis in tumor cells [31]. Furthermore, the high-risk groups, characterized by these CRlncRNAs, showed HLA-related pathways and significant upregulation in Type II IFN Response, which are linked to Treg, macrophages, and CTLs [108–110].

Impact on Immune Response: In addition, COVID-19 and the upregulation of cuproptosis-related genes are linked to immune dysregulation [111]. Immune scoring analysis showed that the high-risk cohort characterized by CRlncRNAs demonstrated an increase in the HLA-related pathways and interferon-gamma (IFN-γ) response. These outcomes suggest that cuproptosis with associated ncRNAs can modulate immune responses in lung cancer patients with COVID-19 [112–114].

## Cuproptosis and drug sensitivity in lung cancer

Cuproptosis-related ncRNAs perform a substantial role in predicting drug sensitivity in bronchogenic carcinoma, influencing responses to various therapies including chemotherapy, targeted therapy, and radiation [3, 39, 43, 115] Table 1.

**Table 1:** Key lncRNAs associated with drug sensitivity and resistance in lung cancer

lncRNA	Role in drug sensitivity	Mechanism	References
MALAT1	Promotes resistance to cisplatin and other chemotherapies	Upregulates MRP1/ABCC1 and MDR1/ABCB1 via STAT3 activation, enhancing drug efflux and reducing drug efficacy	116, 117
HOTAIR	Mediates resistance to chemotherapy and targeted therapies	Recruits PRC2 and LSD1 complexes to modify chromatin, suppressing apoptosis and promoting survival pathways	^116, 118^
H19	Enhances resistance to chemotherapy by regulating apoptosis and drug metabolism	Sponges miR-141, activating the β-catenin pathway and increasing ALDH activity, leading to chemo-resistance	^117, 118^
ANRIL	Associated with resistance to cisplatin and 5-fluorouracil	Upregulates MDR1/ABCB1 and MRP1/ABCC1, reducing drug accumulation and efficacy	^117, 118^
GAS5	Sensitizes cancer cells to chemotherapy by inducing apoptosis	Regulates p53 and E2F1 pathways, promoting apoptosis and reducing cell survival under drug treatment	^116, 119^
MEG3	Enhances sensitivity to chemotherapy by inhibiting cell proliferation and inducing apoptosis	Activates p53, leading to apoptosis and reduced chemo-resistance in NSCLC cells	^116, 119^
UCA1	Promotes resistance to chemotherapy and targeted therapies	Sponges miRNAs, enhancing cell proliferation, migration, and EMT, while inhibiting apoptosis	^119, 120^
TUG1	Contributes to chemo-resistance by regulating cell cycle and apoptosis	Interacts with PRC2, suppressing apoptosis and promoting cell survival under drug treatment	^116, 119^
PVT1	Mediates resistance to cisplatin and other chemotherapies	Upregulates MDR1, MRP, mTOR, and HIF-1α, reducing drug-induced apoptosis	^117^
XIST	Associated with resistance to doxorubicin and other chemotherapies	Sponges miR-124, upregulating SGK1 and reducing drug-induced apoptosis	^117^
LUCAT1	Contributes to cisplatin resistance	Regulates the miR-514a-3p/ULK1 axis. Also represses p21 and p57 expression epigenetically, affecting cell proliferation	^33^
CRNDE	Involved in radioresistance	Targets p21	^121, 122^
PTPRG-AS1	Reduces radiosensitivity of NSCLC cells	Regulates MiR-200c-3p/TCF4	^123, 124^
LINC00205	May affect sensitivity to specific drugs	Correlated with patient survival, with high-risk patients showing different sensitivities to specific drugs	^3^
LINC02635	Part of an eight lncRNA signature associated with prognosis and TIME, this signature can predict drug sensitivity	it is associated with tumor immune microenvironment	^21, 32^

As summarized in Table 1, a network of lncRNAs governs drug sensitivity in lung cancer by targeting crucial processes such as apoptosis (e.g., GAS5 and MEG3), drug efflux pumps (e.g., MALAT1, ANRIL, and PVT1), and DNA damage repair (e.g., CRNDE and PTPRG-AS1). The overarching theme is that these lncRNAs frequently confer resistance by suppressing cell death pathways, thereby directly counteracting the pro-death effects of cuproptosis. This suggests that targeting these specific ncRNAs could re-sensitize tumors to conventional therapies by unleashing cuproptosis.

### Chemotherapy

Cuproptosis-related ncRNAs can predict sensitivity to chemotherapeutic agents like cisplatin. Some studies have shown that high-risk patients, as defined by CRlncRNA signatures, generally exhibit a higher response to chemotherapeutic agents ^20^. For instance, research conducted by Li et al. (2024) suggests that specific lncRNA signatures can assist in identifying patients who are more prone to respond to chemotherapy ^31^. However, some studies indicate that certain lncRNAs may play a role in mediating chemo-resistance in bronchogenic carcinoma cells ^121,122,125^. For example, the lncRNA MALAT1 has been shown to modulate chemo-resistance of bronchogenic carcinoma to cisplatin through Wnt/β-catenin signaling pathway [126–128].

### Targeted therapy

There is a notable association between cuproptosis-related ncRNAs and response to targeted therapies such as EGFR inhibitors. Some studies suggest that high-risk groups might have different sensitivities to specific drugs ^129–131^. For example, a study found that CBX2 is significantly higher in expression in EGFR wild type groups compared to EGFR mutation groups ^57^. Additionally, some CRlncRNA signatures have been used to identify potential drugs that might work well in treating patients with high-risk scores, such as cetuximab and gefitinib ^31^. Another study identified epothilone-b and gemcitabine as potential drugs for patients with elevated risk scores derived from a CRlncRNA signature ^32^.

### Radio-sensitization

Cuproptosis and radio-sensitization are interconnected, with cuproptosis enhancing radiation response and ncRNAs playing a regulatory function in DNA damage repair (DDR)^33^.

Radio-sensitization is the process of enhancing the sensitivity of cancer cells to radiation therapy, and cuproptosis, a Cu-dependent cellular death mechanism, has come forth as a novel approach to achieve this. Cuproptosis is triggered by the accumulation of Cu, which interferes with mitochondrial activity leading to cellular death ^132–134^. Cancer cells, which frequently display disrupted Cu metabolism, are particularly susceptible to cuproptosis, making it a potential target for radio-sensitization ^45^.

LncRNAs have been shown to manage the Cu transporters’ gene regulation as well as other key elements of cuproptosis pathways ^41,42,51^. By modulating these pathways, lncRNAs can influence the sensitivity of malignant cells to both Cu-induced cellular damage and radiation therapy ^135–138^.

Specific lncRNA signatures have been identified as prognostic biomarkers of NSCLC patients following radiation therapy ^33,139,140^.

These signatures can assist in determining which patients stand to gain the most from a combination of cuproptosis and radiation therapy ^33^.

For example, a study developed a 6-CRlncRNAs-model that showed improved accuracy in predicting patient prognosis after radiation compared to traditional clinical factors. This model included lncRNAs like LUCAT1, AC104088.1, HHLA3-AS1, PPP4R3B-DT, AC006042.3, as well as LINC020291 ^33^. High-risk lncRNAs such as HHLA3-AS1, LUCAT1, and LINC02029 were linked to poorer outcomes, while PPP4R3B-DT, AC104088.1, and AC006042.3 were considered protective ^33^.

Moreover, specific lncRNAs can influence the DDR correlated genetic expression, thereby impacting how cancer cells respond to radiation therapy ^89,141,142^. For example, lncRNA PTPRG-AS1 has been found to decrease radiosensitivity in NSCLC cells ^123,143^. Nevertheless, lncRNA CRNDE, through its interaction with PRC2 and targeting of p21, has been shown to enhance radiosensitivity in NSCLC ^122,144^.

It is worth noting that radiation therapy can induce DNA damage, leading to mutations in both cancerous and healthy cells—a factor that is frequently overlooked ^102^. Interestingly, a high TMB is generally accompanied by better responses to both radiation therapy and immunotherapy ^33^. Combining radiotherapy with CRlncRNAs may improve the efficacy of lung cancer treatment by overcoming resistance to radiotherapy and enhancing cell death ^48,145^. In addition, some studies suggest that the integration of cuproptosis induction with immune checkpoint inhibitors can enhance radiosensitivity ^47,146^.

The ncRNA-mediated regulation of cuproptosis is a key determinant of lung cancer's response to therapeutic agents, a feature intrinsically linked to the remodeling of the tumor microenvironment and the acquisition of stem-like properties discussed in the following sections.

## Cuproptosis and tumor microenvironment (TME) remodeling

Cuproptosis-related ncRNAs significantly participate in remodeling the TME via regulating stromal cells, extracellular matrix (ECM), and cancer stemness.

### Stromal cell activation

Cuproptosis-related ncRNAs can impact stromal cells’ behavior inside the TME. For instance, Yang *et al.* (2023) revealed specific CRlncRNAs which can alter the TME in gastric cancer ^147–150^. They can predict the patient survival through an identified signature of six lncRNAs and correlates with variations in immunity stimulation and TME features ^147–150^. Such findings suggest that susceptible subpopulations, defined by these lncRNAs, may exhibit elevated TME scores and could potentially respond better to anti-PD-1 immune checkpoint therapy ^147–150^. Furthermore, another study demonstrated that the lncRNA PVT1 interacts with TME components to promote apoptosis resistance and enhance cancer cell survival ^151–153^. This highlights lncRNAs function in facilitating interaction between tumor cells and their surrounding stromal environment, ultimately influencing tumor progression and treatment outcomes ^21,44^.

### Extracellular matrix remodeling

Cuproptosis-related ncRNAs can impact the ECM, which is the TME core element. Several studies highlight how lncRNAs can modulate the ECM to promote tumor invasion and metastasis ^149,150,154^. For example, it is noted that the ECM, immune cells, and fibroblasts within the TME are main components for lncRNAs expression ^151,154,155^. Some lncRNAs are recognized to interrelate with the ECM, stimulating tumor invasion and metastasis. Furthermore, the TME can affect lncRNA expression, creating a feedback loop where the tumor environment and ncRNA regulation of the ECM influence each other ^44,156,157^.

The cuproptosis-TME remodeling driven by ncRNAs, as described here, creates a protective niche that not fosters immune evasion but also promotes the survival of treatment-resistant cancer stem cells.

## Cuproptosis and stemness

The ncRNAs perform a substantial function in regulating tumor stemness and oncogenic pathways, which significantly contribute to tumor initiation and progression.

Cancer stem cells (CSCs) refer to a subset of tumor cells which are characterized by differentiation capabilities and self-sustenance, accountable for tumorigenesis, metastasis, and resistance to therapies. LncRNAs have been shown to regulate CSC properties, thereby influencing tumor behavior ^158–160^. For instance, some lncRNAs promote cancer stemness by antagonizing the degradation of specific proteins ^158–160^.

LncRNAs can modulate various oncogenic pathways, such as the Wnt/β-catenin signaling pathway, which is crucial for cell proliferation and stemness maintenance ^161,162^. For example, lncRNA JPX regulates the Wnt/β-catenin signaling pathway, therefore affecting tumor growth and metastasis ^163^. Additionally, the lncRNA MALAT1 has been shown to suppress the Wnt/β-catenin signaling pathway in colon cancer cells, suggesting the complex and context-dependent roles of lncRNAs in cancer ^164,165^.

Specific lncRNAs, such as PVT1, have been found to promote cancer progression by influencing the TME. For example, PVT1 can promote pancreatic cancer progression and immune exclusion by interacting with tumor-associated non-myelinating Schwann cells ^151,155,166^.

LncRNAs assist in regulation critical signaling pathways, like the PI3K/AKT/mTOR pathway, which governs cellular metabolism, division, and survival ^167,168^. For instance, the lncRNA H19 influences the PI3K/AKT pathway by acting as a ceRNA for miR-29a, potentially counteracting cuproptosis by promoting cellular survival and proliferation ^167,168^.

The relationship between cuproptosis and stemness is intricate. Research indicates that cancer cells may become more susceptible to Cu-induced stress and cuproptosis when they transition into a more heterogeneous state ^169–171^. In this context, lncRNAs may serve as key targets in cancer-related processes, offering potential for therapeutic strategies designed to induce cuproptosis ^169–171^.

Specific lncRNAs assist in genetic regulation of Cu transporters, which are fundamental for maintaining intracellular Cu balance. Dysregulation of these transporters rendering to storage of Cu, mitochondrial dysfunction and cell death. Additionally, lncRNAs can modulate genes involved in mitochondrial respiration and ROS production ^42,51^.

Certain lncRNAs are linked to autophagy, a cellular mechanism that breaks down and recycles damaged proteins and organelles. For example, the lncRNA HOTAIR inhibits autophagy, potentially exacerbating mitochondrial dysfunction and promoting cuproptosis by preventing the clearance of damaged mitochondria ^88,141^.

Furthermore, certain lncRNAs have been found to be associated with stemness-related properties in lung adenocarcinoma ^20^.

## Therapeutic implications and future directions

**Fig. 3:**
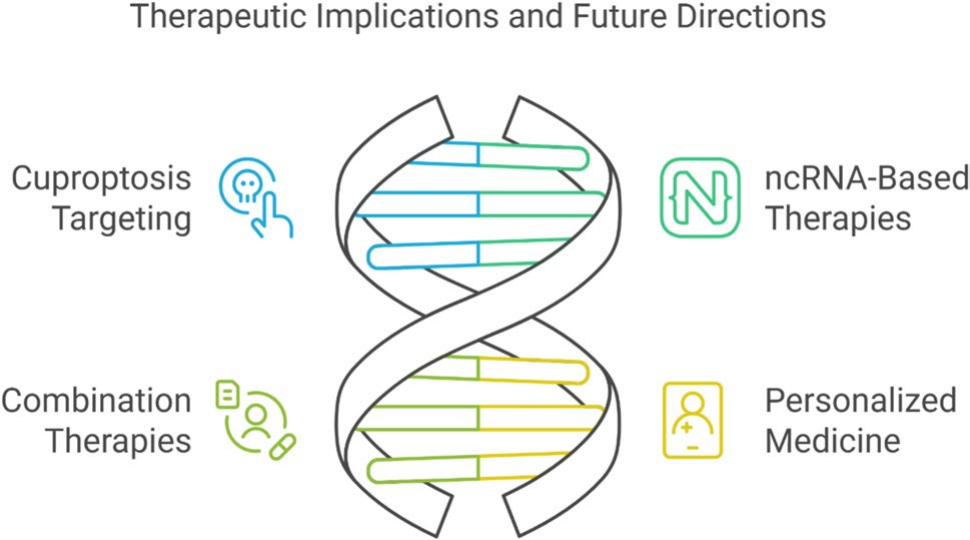
Therapeutic implications and future directions against cuproptosis in lung cancer

The therapeutic potential of targeting cuproptosis pathways, developing ncRNA-based therapeutics, and exploring combination therapies present promising avenues for lung cancer treatment, though challenges and limitations remain Fig. 3.

### Cuproptosis as a therapeutic target

Cuproptosis mechanism is a potential target for tumor treatment due to the increased susceptibility of tumor cells to Cu-stimulated cellular damage ^172–175^. Scientists are creating drugs that induce cuproptosis and agents that modify Cu metabolism to selectively kill cancer cells while minimizing harm to normal tissues ^172–175^. Strategies include using Cu chelators to reduce intracellular Cu levels and creating compounds that specifically target and kill tumor cells. The ability of cuproptosis to overcome resistance to conventional therapies makes it a valuable approach^3^.

### ncRNA-based therapies

Given the role of ncRNAs, particularly lncRNAs, in regulating cuproptosis, there is interest in developing ncRNA-based therapeutics ^176,177^. These therapies aim to modulate cuproptosis pathways by manipulating lncRNA expression or function. Potential approaches include using antisense oligonucleotides (ASOs) to silence oncogenic lncRNAs or employing small molecule inhibitors to block lncRNA activity ^176,177^. For example, personalized medicine could be designed to combat the lncRNA HOTAIR, which is upregulated in malignancies and associated with a bad prognosis, thereby making the cancer more sensitive to cuproptosis ^3^. However, further research is necessary to ensure that targeting specific lncRNAs does not negatively impact normal cellular processes ^176,177^.

### Combination therapies

The integration of cuproptosis modulation with other cancer treatments, like chemotherapy, immunotherapy, and targeted therapy, is being explored ^178–180^. Studies suggest that combining cuproptosis induction with immune checkpoint inhibitors can enhance radiosensitivity ^20^. Furthermore, CRlncRNA signatures have shown the potential to forecast patient response to specific drugs, enabling the personalization of therapeutic approaches ^2,3,181^. For example, high-risk patients with specific lncRNA profiles may exhibit remarkable immunotherapy responses and differing sensitivities to certain antineoplastic drugs ^130,182^. Combining cuproptosis-inducing drugs with conventional chemotherapy or radiation therapy could enhance treatment efficacy ^33^.

### Personalized medicine

Therapeutic strategies that adapt treatment according to the distinct lncRNA expression profiles of a patient's cancer could be developed 176, 177. This personalized approach may enable the design of targeted therapies that inhibit specific lncRNAs, thereby increasing the tumor's vulnerability to cuproptosis 176, 177. Additionally, further research into the role of Cu metabolism and cuproptosis across different types of tumors, as well as the intricate interplay between these processes, ncRNAs, and the immune system, is crucial to enhance treatment outcomes ^156,178,183^.

### Challenges and opportunities

Despite its promise, several challenges remain to be addressed ^156,178,183^. More studies are required to completely understand the processes via which lncRNAs regulate cuproptosis ^14,157^. Additionally, the specificity of ncRNA-based therapies must be enhanced to reduce off-target effects. Future studies should focus on identifying specific lncRNAs that can serve as biomarkers for early cancer detection, prognosis assessment, and tracking disease progression ^156,178,183^. It is equally crucial to develop sophisticated delivery mechanisms to enable the effective and targeted distribution of therapeutic ncRNAs to cancerous cells ^176,177,184^. A deeper understanding of how Cu accumulation triggers mitochondrial dysfunction and cell death is essential to advance this field ^157,185^.

## Conclusion

Cuproptosis, a unique type of regulated cell suicide, may be regulated by ncRNAs and epigenetic modifications and implicated in lung cancer progression. These ncRNAs influence tumorigenesis, immune evasion, drug resistance, and tumor microenvironment remodeling, making them potential diagnostic biological markers as well as pharmaceutical targets.

Targeting cuproptosis pathways offers promising therapeutic strategies, particularly through ncRNA-based treatments and personalized medicine. Future research should focus on refining these interventions and validating biomarkers for clinical applications. Advancements in this field could significantly improve lung cancer prognosis and treatment outcomes.

### Limitations and evidence gaps

(i) Distinguishes bioinformatic associations from experimental causality.
(ii) Notes the predominance of TCGA-based signatures and limited external validation.
(iii) Discusses context dependence across tumor histologies.

## Data Availability

No datasets were generated or analysed during the current study.

## References

[1] Ye W, Huang Y, Li X. Cuproptosis-related gene signatures for predicting prognosis of lung adenocarcinoma. Medicine. 2022;101:E30446. 36221373 10.1097/MD.0000000000030446 PMC9542578

[2] Wang F, Lin H, Su Q, Li C. Cuproptosis-related lncRNA predict prognosis and immune response of lung adenocarcinoma. World J Surg Oncol. 2022. 10.1186/s12957-022-02727-7. 36050740 10.1186/s12957-022-02727-7 PMC9434888

[3] Bhat AA, et al. lncRNAs as prognostic markers and therapeutic targets in cuproptosis-mediated cancer. Clin Exp Med. 2024;24(1):226. 39325172 10.1007/s10238-024-01491-0 PMC11427524

[4] Ettinger DS, et al. Non-small cell lung cancer, version 6.2015. J Natl Compr Canc Netw. 2015;13:515–24. 25964637 10.6004/jnccn.2015.0071

[5] Hou C, et al. A cuproptosis-associated long non-coding RNA signature for the prognosis and immunotherapy of lung squamous cell carcinoma. Biomol Biomed. 2023;23:624–33. 36724022 10.17305/bb.2022.8481 PMC10351099

[6] Yu S, et al. A cuproptosis-related lncRNA signature for predicting prognosis and immunotherapy response of lung adenocarcinoma. Hereditas. 2023. 10.1186/s41065-023-00293-w. 37482612 10.1186/s41065-023-00293-w PMC10364405

[7] Tsvetkov P, et al. Copper induces cell death by targeting lipoylated TCA cycle proteins. Science. 2022;375:1254–61. 35298263 10.1126/science.abf0529 PMC9273333

[8] Bisaglia M, Bubacco L. Copper ions and Parkinson’s disease: why is homeostasis so relevant? Biomolecules. 2020. 10.3390/biom10020195. 32013126 10.3390/biom10020195 PMC7072482

[9] Wang Y, Xiao X, Li Y. Construction and validation of a cuproptosis-related lncRNA signature for the prediction of the prognosis of LUAD and LUSC. Sci Rep. 2023;13:2477. 36774418 10.1038/s41598-023-29719-1 PMC9922262

[10] Chen L, Min J, Wang F. Copper homeostasis and cuproptosis in health and disease. Signal Transduct Target Ther. 2022. 10.1038/s41392-022-01229-y. 36414625 10.1038/s41392-022-01229-y PMC9681860

[11] Wang X, Jing H, Li H. A novel cuproptosis-related lncRNA signature to predict prognosis and immune landscape of lung adenocarcinoma. Transl Lung Cancer Res. 2023;12:230–46. 36895935 10.21037/tlcr-22-500 PMC9989802

[12] Tang D, Chen X, Kroemer G. Cuproptosis: a copper-triggered modality of mitochondrial cell death. Cell Res. 2022. 10.1038/s41422-022-00653-7. 35354936 10.1038/s41422-022-00653-7 PMC9061796

[13] Cheng B, et al. Cuproptosis illustrates tumor micro-environment features and predicts prostate cancer therapeutic sensitivity and prognosis. Life Sci. 2023. 10.1016/j.lfs.2023.121659. 37011878 10.1016/j.lfs.2023.121659

[14] Di H, et al. A novel prognostic signature for lung adenocarcinoma based on cuproptosis-related lncRNAs: a review. Medicine (Baltimore). 2022;101:E31924. 36626411 10.1097/MD.0000000000031924 PMC9750635

[15] Jiang Y, Huo Z, Qi X, Zuo T, Wu Z. Copper-induced tumor cell death mechanisms and antitumor theragnostic applications of copper complexes. Nanomedicine (Lond). 2022;17:303–24. 35060391 10.2217/nnm-2021-0374

[16] Li Y. Copper homeostasis: emerging target for cancer treatment. IUBMB Life. 2020;72:1900–8. 32599675 10.1002/iub.2341

[17] Katsura C, Ogunmwonyi I, Kankam HKN, Saha S. Breast cancer: presentation, investigation and management. Br J Hosp Med (Lond). 2022. 10.12968/hmed.2021.0459. 35243878 10.12968/hmed.2021.0459

[18] Wang Y, Zhang L, Zhou F. Cuproptosis: a new form of programmed cell death. Cell Mol Immunol. 2022;19:867–8. 35459854 10.1038/s41423-022-00866-1 PMC9338229

[19] Zhao Q, Qi T. The implications and prospect of cuproptosis-related genes and copper transporters in cancer progression. Front Oncol. 2023. 10.3389/fonc.2023.1117164. 36925927 10.3389/fonc.2023.1117164 PMC10011146

[20] Wang Z, Yao J, Dong T, Niu X. Definition of a novel cuproptosis-relevant lncrna signature for uncovering distinct survival, genomic alterations, and treatment implications in lung adenocarcinoma. J Immunol Res. 2022;2022(1):2756611. 36281357 10.1155/2022/2756611 PMC9587678

[21] Song J, et al. Construction and validation of a cuproptosis-related lncRNA prognosis signature in bladder carcinoma. J Cancer Res Clin Oncol. 2023. 10.1007/S00432-023-05013-5. 37354222 10.1007/s00432-023-05013-5 PMC11797366

[22] Ma S, et al. A cuproptosis-related long non-coding RNA signature to predict the prognosis and immune microenvironment characterization for lung adenocarcinoma. Transl Lung Cancer Res. 2022;11:2079–93. 36386454 10.21037/tlcr-22-660 PMC9641048

[23] Ruiz-Cordero R, Devine WP. Targeted therapy and checkpoint immunotherapy in lung cancer. Surg Pathol Clin. 2020;13:17–33. 32005431 10.1016/j.path.2019.11.002

[24] Chen Q, et al. Cuproptosis-related LncRNA signature for predicting prognosis of hepatocellular carcinoma: a comprehensive analysis. Dis Mark. 2022;2022:3265212. 10.1155/2022/3265212 PMC9705118 36452343

[25] Yu Z, et al. Blockage of SLC31A1-dependent copper absorption increases pancreatic cancer cell autophagy to resist cell death. Cell Prolif. 2019. 10.1111/cpr.12568. 30706544 10.1111/cpr.12568 PMC6496122

[26] Huang H, et al. Deciphering the role of cuproptosis-related lncRNAs in shaping the lung cancer immune microenvironment: a comprehensive prognostic model. J Cell Mol Med. 2024. 10.1111/jcmm.18519. 38973477 10.1111/jcmm.18519 PMC11228428

[27] Yang L, Cui Y, Liang L, Lin J. Significance of cuproptosis-related lncRNA signature in LUAD prognosis and immunotherapy: a machine learning approach. Thorac Cancer. 2023;14:1451–66. 37076991 10.1111/1759-7714.14888 PMC10234775

[28] Duma N, Santana-Davila R, Molina JR. Non-small cell lung cancer: epidemiology, screening, diagnosis, and treatment. Mayo Clin Proc. 2019;94:1623–40. 31378236 10.1016/j.mayocp.2019.01.013

[29] Yalimaimaiti S, et al. Establishment of a prognostic signature for lung adenocarcinoma using cuproptosis-related lncRNAs. BMC Bioinformatics. 2023;24:81. 36879187 10.1186/s12859-023-05192-5 PMC9990240

[30] Pang L, et al. Development and validation of cuproptosis-related lncRNA signatures for prognosis prediction in colorectal cancer. BMC Med Genomics. 2023;16:58. 36949429 10.1186/s12920-023-01487-x PMC10031908

[31] Li J, et al. Cuproptosis-associated lncRNA impact prognosis in patients with non-small cell lung cancer co-infected with COVID-19. J Cell Mol Med. 2024. 10.1111/jcmm.70059. 39228012 10.1111/jcmm.70059 PMC11371660

[32] Ma C, Gu Z, Ding W, Li F, Yang Y. Crosstalk between copper homeostasis and cuproptosis reveals a LncRNA signature to prognosis prediction, immunotherapy personalization, and agent selection for patients with lung adenocarcinoma. Aging (Albany NY). 2023;15(22):13504. 38011277 10.18632/aging.205281 PMC10713389

[33] Xu Q, Liu T, Wang J. Radiosensitization-related cuproptosis lncRNA signature in non-small cell lung cancer. Genes. 2022. 10.3390/genes13112080. 36360316 10.3390/genes13112080 PMC9690519

[34] Yoo S, et al. Integrative network analysis of early-stage lung adenocarcinoma identifies aurora kinase inhibition as interceptor of invasion and progression. Nat Commun. 2022. 10.1038/s41467-022-29230-7. 35332150 10.1038/s41467-022-29230-7 PMC8948234

[35] Wu J, et al. Cuproptosis: mechanism, role, and advances in urological malignancies. Med Res Rev. 2024;44:1662–82. 38299968 10.1002/med.22025

[36] Xiong C, Ling H, Hao Q, Zhou X. Cuproptosis: p53-regulated metabolic cell death? Cell Death Differ. 2023;30:876–84. 36755067 10.1038/s41418-023-01125-0 PMC10070433

[37] Kahlson MA, Dixon SJ. Copper-induced cell death. Science. 2022;375:1231–2. 35298241 10.1126/science.abo3959

[38] Liu H, Tang T. Pan-cancer genetic analysis of cuproptosis and copper metabolism-related gene set. Front Oncol. 2022. 10.3389/fonc.2022.952290. 36276096 10.3389/fonc.2022.952290 PMC9582932

[39] Springer C, Humayun D, Skouta R. Cuproptosis: unraveling the mechanisms of copper-induced cell death and its implication in cancer therapy. Cancers (Basel). 2024. 10.3390/cancers16030647. 38339398 10.3390/cancers16030647 PMC10854864

[40] Singh AK, et al. Current therapeutic modalities and chemopreventive role of natural products in liver cancer: progress and promise. World J Hepatol. 2023;15:1–18. 36744169 10.4254/wjh.v15.i1.1 PMC9896505

[41] Gao L, Zhang A. Copper-instigated modulatory cell mortality mechanisms and progress in oncological treatment investigations. Front Immunol. 2023. 10.3389/fimmu.2023.1236063. 37600774 10.3389/fimmu.2023.1236063 PMC10433393

[42] Vo TTT, et al. The crosstalk between copper-induced oxidative stress and cuproptosis: a novel potential anticancer paradigm. Cell Commun Signal. 2024. 10.1186/s12964-024-01726-3. 38970072 10.1186/s12964-024-01726-3 PMC11225285

[43] Ma C, Li F, Gu Z, Yang Y, Qi Y. A novel defined risk signature of cuproptosis-related long non-coding RNA for predicting prognosis, immune infiltration, and immunotherapy response in lung adenocarcinoma. Front Pharmacol. 2023. 10.3389/fphar.2023.1146840. 37670938 10.3389/fphar.2023.1146840 PMC10475834

[44] Yang T, Chen M, Chen T, Thakur A. Expression of the copper transporters hCtr1, ATP7A and ATP7B is associated with the response to chemotherapy and survival time in patients with resected non-small cell lung cancer. Oncol Lett. 2015;10:2584–90. 26622894 10.3892/ol.2015.3531 PMC4580033

[45] Abdullah KM, et al. Copper metabolism and cuproptosis in human malignancies: unraveling the complex interplay for therapeutic insights. Heliyon. 2024. 10.1016/j.heliyon.2024.e27496. 38486750 10.1016/j.heliyon.2024.e27496 PMC10938126

[46] Guo CY, Sun L, Chen XP, Zhang DS. Oxidative stress, mitochondrial damage and neurodegenerative diseases. Neural Regen Res. 2013;8:2003–14. 25206509 10.3969/j.issn.1673-5374.2013.21.009 PMC4145906

[47] Zhou S, et al. Identification of a pyroptosis-related lncRNA signature in the regulation of prognosis, metabolism signals and immune infiltration in lung adenocarcinoma. Front Endocrinol (Lausanne). 2022. 10.3389/fendo.2022.964362. 36034461 10.3389/fendo.2022.964362 PMC9401518

[48] Oliveri V. Selective targeting of cancer cells by copper ionophores: an overview. Front Mol Biosci. 2022;9:841814. 35309510 10.3389/fmolb.2022.841814 PMC8931543

[49] Lelièvre P, Sancey L, Coll JL, Deniaud A, Busser B. The multifaceted roles of copper in cancer: a trace metal element with dysregulated metabolism, but also a target or a bullet for therapy. Cancers (Basel). 2020;12:1–25. 10.3390/cancers12123594 PMC7760327 33271772

[50] McCabe EM, Rasmussen TP. LncRNA involvement in cancer stem cell function and epithelial-mesenchymal transitions. Semin Cancer Biol. 2021;75:38–48. 33346133 10.1016/j.semcancer.2020.12.012

[51] Zhu Z, et al. Comprehensive analysis of cuproptosis-related lncRNAs to predict prognosis and immune infiltration characteristics in colorectal cancer. Front Genet. 2022;13:984743. 36467996 10.3389/fgene.2022.984743 PMC9712968

[52] Liu L, et al. Long non-coding RNA OGFRP1 regulates cell proliferation and ferroptosis by miR-299-3p/SLC38A1 axis in lung cancer. Anticancer Drugs. 2022;33:826–39. 36066402 10.1097/CAD.0000000000001328

[53] Xue Y, et al. Long noncoding RNAs *PTPRG* antisense RNA 1 targets cyclin D1 to facilitate cell proliferation in lung adenocarcinoma. Cancer Biother Radiopharm. 2024. 10.1089/cbr.2021.0168. 34767727 10.1089/cbr.2021.0168

[54] Lee SS, Cheah YK. The interplay between microRNAs and cellular components of tumour microenvironment (TME) on non-small-cell lung cancer (NSCLC) progression. J Immunol Res. 2019;2019(1):3046379. 30944831 10.1155/2019/3046379 PMC6421779

[55] Guo C, et al. Overexpression of *HOXA10* is associated with unfavorable prognosis of acute myeloid leukemia. BMC Cancer. 2020. 10.1186/s12885-020-07088-6. 32571260 10.1186/s12885-020-07088-6 PMC7310421

[56] Briata P, Gherzi R. Long non-coding RNA-ribonucleoprotein networks in the post-transcriptional control of gene expression. Non-coding RNA. 2020. 10.3390/ncrna6030040. 32957640 10.3390/ncrna6030040 PMC7549350

[57] Wu J, Fu G, Luo C, Chen L, Liu Q. Cuproptosis-related ceRNA axis triggers cell proliferation and cell cycle through CBX2 in lung adenocarcinoma. BMC Pulm Med. 2024. 10.1186/s12890-024-02887-0. 38355480 10.1186/s12890-024-02887-0 PMC10865584

[58] Wu X, Sui Z, Zhang H, Wang Y, Yu Z. Integrated analysis of lncRNA-mediated ceRNA network in Lung Adenocarcinoma. Front Oncol. 2020. 10.3389/fonc.2020.554759. 33042838 10.3389/fonc.2020.554759 PMC7523091

[59] Tiansheng G, et al. lncRNA metastasis-associated lung adenocarcinoma transcript 1 promotes proliferation and invasion of non-small cell lung cancer cells via down-regulating miR-202 expression. Cell J. 2020;22:375–85. 31863664 10.22074/cellj.2020.6837 PMC6947012

[60] Gao C, et al. Development and validation of the potential biomarkers based on m6A-related lncRNAs for the predictions of overall survival in the lung adenocarcinoma and differential analysis with cuproptosis. BMC Bioinformatics. 2022. 10.1186/s12859-022-04869-7. 35941550 10.1186/s12859-022-04869-7 PMC9358839

[61] Lu J, et al. Emerging roles of liquid-liquid phase separation in cancer: from protein aggregation to immune-associated signaling. Front Cell Dev Biol. 2021. 10.3389/fcell.2021.631486. 34235141 10.3389/fcell.2021.631486 PMC8255971

[62] Somasundaram K, Gupta B, Jain N, Jana S. LncRNAs divide and rule: the master regulators of phase separation. Front Genet. 2022. 10.3389/fgene.2022.930792. 36035193 10.3389/fgene.2022.930792 PMC9399341

[63] Chen W, et al. Machine learning diagnostic model for hepatocellular carcinoma based on liquid-liquid phase separation and ferroptosis-related genes. Turk J Gastroenterol. 2025;36:89–99. 10.5152/tjg.2024.24101 PMC11843271 39635757

[64] Elguindy MM, Mendell JT. NORAD-induced Pumilio phase separation is required for genome stability. Nature. 2021;595:303–8. 34108682 10.1038/s41586-021-03633-w PMC8266761

[65] Roden C, Gladfelter AS. RNA contributions to the form and function of biomolecular condensates. Nat Rev Mol Cell Biol. 2021;22:183–95. 32632317 10.1038/s41580-020-0264-6 PMC7785677

[66] Li C, et al. LncRNA SNHG9 is a prognostic biomarker and correlated with immune infiltrates in prostate cancer. Transl Androl Urol. 2021;10:215–26. 33532311 10.21037/tau-20-1134 PMC7844523

[67] Xia Y, et al. Long noncoding RNA H19: functions and mechanisms in regulating programmed cell death in cancer. Cell Death Discov. 2024. 10.1038/s41420-024-01832-8. 38355574 10.1038/s41420-024-01832-8 PMC10866971

[68] Chen L, et al. Chrysin induced cell apoptosis through H19/let-7a/COPB2 axis in gastric cancer cells and inhibited tumor growth. Front Oncol. 2021. 10.3389/fonc.2021.651644. 34150620 10.3389/fonc.2021.651644 PMC8209501

[69] Wang C, et al. Stress induces dynamic, cytotoxicity-antagonizing TDP-43 nuclear bodies via paraspeckle lncRNA NEAT1-mediated liquid-liquid phase separation. Mol Cell. 2020;79:443-458.e7. 32649883 10.1016/j.molcel.2020.06.019

[70] Zhao W, Zhu X, Jin Q, Lin B, Ji R. The lncRNA NEAT1/miRNA-766-5p/E2F3 regulatory axis promotes prostate cancer progression. J Oncol. 2022;2022(1):1866972. 35237319 10.1155/2022/1866972 PMC8885187

[71] Cong Y, Li N, Zhang Z, Shang Y, Zhao H. Cuproptosis: molecular mechanisms, cancer prognosis, and therapeutic applications. J Transl Med. 2025. 10.1186/s12967-025-06121-1. 39844182 10.1186/s12967-025-06121-1 PMC11752808

[72] Li RH, et al. A phosphatidic acid-binding lncRNA SNHG9 facilitates LATS1 liquid–liquid phase separation to promote oncogenic YAP signaling. Cell Res. 2021;31:1088–105. 34267352 10.1038/s41422-021-00530-9 PMC8486796

[73] Liu JL, Gall JG. U bodies are cytoplasmic structures that contain uridine-rich small nuclear ribonucleoproteins and associate with P bodies. Proc Natl Acad Sci U S A. 2007;104:11655–9. 17595295 10.1073/pnas.0704977104 PMC1899408

[74] Huo X, et al. The nuclear matrix protein SAFB cooperates with major satellite RNAs to stabilize heterochromatin architecture partially through phase separation. Mol Cell. 2020;77:368-383.e7. 31677973 10.1016/j.molcel.2019.10.001

[75] Klein IA, et al. Partitioning of cancer therapeutics in nuclear condensates. Science. 2020;1979(368):1386–92. 10.1126/science.aaz4427 PMC7735713 32554597

[76] Li K, et al. Insights into the functions of LncRNAs in drosophila. Int J Mol Sci. 2019. 10.3390/ijms20184646. 31546813 10.3390/ijms20184646 PMC6770079

[77] Shi J, et al. Nop53 undergoes liquid-liquid phase separation and promotes tumor radio-resistance. Cell Death Discov. 2022. 10.1038/s41420-022-01226-8. 36316314 10.1038/s41420-022-01226-8 PMC9622906

[78] Decker CJ, Parker R. P-bodies and stress granules: possible roles in the control of translation and mRNA degradation. Cold Spring Harbor Perspect Biol. 2012;4(9):a012286. 10.1101/cshperspect.a012286 PMC3428773 22763747

[79] Deberardinis RJ, Cheng T. Q’s next: the diverse functions of glutamine in metabolism, cell biology and cancer. Oncogene. 2010;29:313–24. 19881548 10.1038/onc.2009.358 PMC2809806

[80] Geng C, et al. SPOP regulates prostate epithelial cell proliferation and promotes ubiquitination and turnover of c-MYC oncoprotein. Oncogene. 2017;36:4767–77. 28414305 10.1038/onc.2017.80 PMC5887163

[81] Su Y, Maimaitiyiming Y, Wang L, Cheng X, Hsu CH. Modulation of phase separation by RNA: a glimpse on N6-methyladenosine modification. Front Cell Dev Biol. 2021. 10.3389/fcell.2021.786454. 34957114 10.3389/fcell.2021.786454 PMC8703171

[82] El-Ashmawy NE, Khedr EG, Abo-Saif MA, Hamouda SM. Cuproptosis regulation by long noncoding RNAs: mechanistic insights and clinical implications in cancer. Arch Biochem Biophys. 2025. 10.1016/j.abb.2025.110324. 39900259 10.1016/j.abb.2025.110324

[83] Lu Y, et al. Cuproptosis-related lncRNAs emerge as a novel signature for predicting prognosis in prostate carcinoma and functional experimental validation. Front Immunol. 2024. 10.3389/fimmu.2024.1471198. 39530098 10.3389/fimmu.2024.1471198 PMC11550951

[84] Mahmoudi Z, et al. Efficacy of DMARDs and methylprednisolone treatment on the gene expression levels of HSPA5, MMD, and non-coding RNAs MALAT1, H19, miR-199a-5p, and miR-1-3p, in patients with rheumatoid arthritis. Int Immunopharmacol. 2022. 10.1016/j.intimp.2022.108878. 35623291 10.1016/j.intimp.2022.108878

[85] Wang J, et al. Progress in the research of cuproptosis and possible targets for cancer therapy. World J Clin Oncol. 2023;14:324–34. 37771632 10.5306/wjco.v14.i9.324 PMC10523190

[86] Feng Q, et al. Research progress on cuproptosis in cancer. Front Pharmacol. 2024. 10.3389/fphar.2024.1290592. 38357312 10.3389/fphar.2024.1290592 PMC10864558

[87] Wei M, Lu L, Luo Z, Ma J, Wang J. Prognostic analysis of hepatocellular carcinoma based on cuproptosis -associated lncRNAs. BMC Gastroenterol. 2024. 10.1186/s12876-024-03219-6. 38654165 10.1186/s12876-024-03219-6 PMC11040954

[88] Kumar A, et al. Targeting autophagy using long non-coding RNAs (LncRNAs): new landscapes in the arena of cancer therapeutics. Cells. 2023. 10.3390/cells12050810. 36899946 10.3390/cells12050810 PMC10000689

[89] Lin T, et al. Emerging roles of p53 related lncRNAs in cancer progression: a systematic review. Int J Biol Sci. 2019;15:1287–98. 31223287 10.7150/ijbs.33218 PMC6567798

[90] Shi Y, Norberg E, Vakifahmetoglu-Norberg H. Mutant p53 as a regulator and target of autophagy. Front Oncol. 2021. 10.3389/fonc.2020.607149. 33614491 10.3389/fonc.2020.607149 PMC7886977

[91] Li A, et al. Mitochondrial autophagy: molecular mechanisms and implications for cardiovascular disease. Cell Death Dis. 2022. 10.1038/s41419-022-04906-6. 35534453 10.1038/s41419-022-04906-6 PMC9085840

[92] Wu L, et al. A novel cuproptosis-related lncRNAs signature predicts prognosis in bladder cancer. Aging (Albany NY). 2023. 10.18632/aging.204861. 37424068 10.18632/aging.204861 PMC10373974

[93] Yuan HJ, Xue YT, Liu Y. Cuproptosis, the novel therapeutic mechanism for heart failure: a narrative review. Cardiovasc Diagn Ther. 2022;12:681–707. 36329965 10.21037/cdt-22-214 PMC9622411

[94] Yang Y, et al. Exploring cuproptosis as a mechanism and potential intervention target in cardiovascular diseases. Front Pharmacol. 2023. 10.3389/fphar.2023.1229297. 37637426 10.3389/fphar.2023.1229297 PMC10450925

[95] Guo J, Sun Y, Liu G. The mechanism of copper transporters in ovarian cancer cells and the prospect of cuproptosis. J Inorg Biochem. 2023. 10.1016/j.jinorgbio.2023.112324. 37481825 10.1016/j.jinorgbio.2023.112324

[96] Lei B, et al. A novel cuproptosis-associated LncRNA model predicting prognostic and immunotherapy response for glioma. Discover Oncol. 2025. 10.1007/s12672-025-02912-6. 10.1007/s12672-025-02912-6 PMC12165929 40512434

[97] Zhang P, et al. Cuproptosis-related lncRNA signatures: predicting prognosis and evaluating the tumor immune microenvironment in lung adenocarcinoma. Front Oncol. 2023;12:1088931. 36733364 10.3389/fonc.2022.1088931 PMC9887198

[98] Li B, Cui Y, Diehn M, Li R. Development and validation of an individualized immune prognostic signature in early-stage nonsquamous non-small cell lung cancer. JAMA Oncol. 2017;3:1529–37. 28687838 10.1001/jamaoncol.2017.1609 PMC5710196

[99] Zhang C, et al. Genomic landscape and immune microenvironment features of preinvasive and early invasive lung adenocarcinoma. J Thorac Oncol. 2019;14:1912–23. 31446140 10.1016/j.jtho.2019.07.031 PMC6986039

[100] Brahmer J, et al. Nivolumab versus docetaxel in advanced squamous-cell non-small-cell lung cancer. N Engl J Med. 2015;373:123–35. 26028407 10.1056/NEJMoa1504627 PMC4681400

[101] Garon EB, et al. Pembrolizumab for the treatment of non-small-cell lung cancer. N Engl J Med. 2015;372:2018–28. 25891174 10.1056/NEJMoa1501824

[102] Li L, et al. Bioinformatics construction and experimental validation of a cuproptosis-related lncRNA prognostic model in lung adenocarcinoma for immunotherapy response prediction. Sci Rep. 2023. 10.1038/s41598-023-29684-9. 36774446 10.1038/s41598-023-29684-9 PMC9922258

[103] Zhang M, et al. LncRNA GATA3-AS1 facilitates tumour progression and immune escape in triple-negative breast cancer through destabilization of GATA3 but stabilization of PD-L1. Cell Prolif. 2020. 10.1111/cpr.12855. 32687248 10.1111/cpr.12855 PMC7507373

[104] Neviani P, et al. Natural killer-derived exosomal miR-186 inhibits neuroblastoma growth and immune escape mechanisms. Cancer Res. 2019;79:1151–64. 30541743 10.1158/0008-5472.CAN-18-0779 PMC6428417

[105] Narayanan IG, Natarajan SK. Peptides derived from histidine and methionine-rich regions of copper transporter 1 exhibit anti-angiogenic property by chelating extracellular Cu. Chem Biol Drug Des. 2018;91:797–804. 29134764 10.1111/cbdd.13145

[106] Denoyer D, Clatworthy SAS, Cater MA. Copper complexes in cancer therapy. Met Ions Life Sci. 2018;18:469–506. 10.1515/9783110470734-022 29394035

[107] Taghiloo S, Norozi S, Asgarian-Omran H. The effects of PI3K/Akt/mTOR signaling pathway inhibitors on the expression of immune checkpoint ligands in acute myeloid leukemia cell line. Iran J Allergy Asthma Immunol. 2022;21:178–88. 35490271 10.18502/ijaai.v21i2.9225

[108] Shapouri-Moghaddam A, et al. Macrophage plasticity, polarization, and function in health and disease. J Cell Physiol. 2018;233:6425–40. 29319160 10.1002/jcp.26429 PMC13482560

[109] Hepatocyte growth factor production by neutrophils infiltrating bronchioloalveolar subtype pulmonary adenocarcinoma: role in tumor progression and death - PubMed. https://pubmed.ncbi.nlm.nih.gov/12649206/. 12649206

[110] Jensen HK, et al. Presence of intratumoral neutrophils is an independent prognostic factor in localized renal cell carcinoma. J Clin Oncol. 2009;27:4709–17. 19720929 10.1200/JCO.2008.18.9498

[111] Cupp MA, et al. Neutrophil to lymphocyte ratio and cancer prognosis: an umbrella review of systematic reviews and meta-analyses of observational studies. BMC Med. 2020. 10.1186/s12916-020-01817-1. 33213430 10.1186/s12916-020-01817-1 PMC7678319

[112] Dong L, et al. Functional differentiation and regulation of follicular T helper cells in inflammation and autoimmunity. Immunology. 2021;163:19–32. 33128768 10.1111/imm.13282 PMC8044332

[113] Zheng R, et al. COVID-19-associated coagulopathy: thromboembolism prophylaxis and poor prognosis in ICU. Exp Hematol Oncol. 2021. 10.1186/s40164-021-00202-9. 33522958 10.1186/s40164-021-00202-9 PMC7848868

[114] Sette A, Crotty S. Adaptive immunity to SARS-CoV-2 and COVID-19. Cell. 2021;184:861–80. 33497610 10.1016/j.cell.2021.01.007 PMC7803150

[115] Zhong G, et al. Cuproptosis is involved in copper-induced hepatotoxicity in chickens. Sci Total Environ. 2023. 10.1016/j.scitotenv.2023.161458. 36621474 10.1016/j.scitotenv.2023.161458

[116] La Montagna M, Ginn L, Garofalo M. Mechanisms of drug resistance mediated by long non-coding RNAs in non-small-cell lung cancer. Cancer Gene Ther. 2020;28:175–87. 32843741 10.1038/s41417-020-00214-3

[117] Liu K, et al. Long non-coding RNAs regulate drug resistance in cancer. Mol Cancer. 2020;19:1–13. 32164712 10.1186/s12943-020-01162-0 PMC7066752

[118] Alnefaie GO. A review of the complex interplay between chemoresistance and lncRNAs in lung cancer. J Transl Med. 2024;22:1–16. 39639388 10.1186/s12967-024-05877-2 PMC11619437

[119] Cao Z, et al. The roles of long non-coding RNAs in lung cancer. J Cancer. 2022;13:174–83. 34976181 10.7150/jca.65031 PMC8692699

[120] Hu Q, Ma H, Chen H, Zhang Z, Xue Q. LncRNA in tumorigenesis of non-small-cell lung cancer: From bench to bedside. Cell Death Discovery. 2022;8:1–9. 35963868 10.1038/s41420-022-01157-4 PMC9376075

[121] Chen M, et al. A novel biosensor for the ultrasensitive detection of the lncRNA biomarker MALAT1 in non-small cell lung cancer. Sci Rep. 2021. 10.1038/s41598-021-83244-7. 33574438 10.1038/s41598-021-83244-7 PMC7878801

[122] Zhang M, et al. Long noncoding RNA CRNDE/PRC2 participated in the radiotherapy resistance of human lung adenocarcinoma through targeting p21 expression. Oncol Res. 2018;26:1245–55. 28550688 10.3727/096504017X14944585873668 PMC7844700

[123] Ma Q, et al. Long noncoding RNA *PTPRG* antisense RNA 1 reduces radiosensitivity of nonsmall cell lung cancer cells via regulating miR-200c-3p/TCF4. Technol Cancer Res Treat. 2020. 10.1177/1533033820942615. 33174523 10.1177/1533033820942615 PMC7672737

[124] Reck M, Remon J, Hellmann MD. First-line immunotherapy for non-small-cell lung cancer. J Clin Oncol. 2022;40:586–97. 34985920 10.1200/JCO.21.01497

[125] Liu B, Zhao Y, Yang S. An autophagy-related long non-coding RNA prognostic signature for patients with lung squamous carcinoma based on bioinformatics analysis. Int J Gen Med. 2021;14:6621–37. 34675625 10.2147/IJGM.S331327 PMC8520473

[126] Chen W, et al. MALAT1-miR-101-SOX9 feedback loop modulates the chemo-resistance of lung cancer cell to DDP via Wnt signaling pathway. Oncotarget. 2017;8:94317–29. 29212230 10.18632/oncotarget.21693 PMC5706876

[127] Xu M, Mu J, Wang J, Zhou Q, Wang J. Construction and validation of a cuproptosis-related lncRNA signature as a novel and robust prognostic model for colon adenocarcinoma. Front Oncol. 2022;12:961213. 35965536 10.3389/fonc.2022.961213 PMC9367690

[128] Wang M, et al. Cuproptosis: emerging biomarkers and potential therapeutics in cancers. Front Oncol. 2023. 10.3389/fonc.2023.1288504. 38023234 10.3389/fonc.2023.1288504 PMC10662309

[129] Fazel SS, et al. Barriers and facilitators for the safe handling of antineoplastic drugs. J Oncol Pharm Pract. 2022;28:1709–21. 34612752 10.1177/10781552211040176

[130] Lima HRS, et al. Electrochemical sensors and biosensors for the analysis of antineoplastic drugs. Biosens Bioelectron. 2018;108:27–37. 29494885 10.1016/j.bios.2018.02.034

[131] Wang Y, et al. A novel risk model construction and immune landscape analysis of gastric cancer based on cuproptosis-related long noncoding RNAs. Front Oncol. 2022;12:1015235. 36387229 10.3389/fonc.2022.1015235 PMC9643840

[132] Wang Z, et al. Regulatory roles of copper metabolism and cuproptosis in human cancers. Front Oncol. 2023. 10.3389/fonc.2023.1123420. 37035162 10.3389/fonc.2023.1123420 PMC10076572

[133] Liang XR, et al. Cell cycle-related lncRNAs as innovative targets to advance cancer management. Cancer Manag Res. 2023;15:547–61. 37426392 10.2147/CMAR.S407371 PMC10327678

[134] Luan M, et al. Mechanism of metal ion-induced cell death in gastrointestinal cancer. Biomed Pharmacother. 2024. 10.1016/j.biopha.2024.116574. 38593706 10.1016/j.biopha.2024.116574

[135] Bai X, et al. Cuproptosis-related lncRNA signature as a prognostic tool and therapeutic target in diffuse large B cell lymphoma. Sci Rep. 2024. 10.1038/s41598-024-63433-w. 38839842 10.1038/s41598-024-63433-w PMC11153514

[136] Chen Y, Tang J, Chen L, Chen J. Novel cuproptosis-related lncRNAs can predict the prognosis of patients with multiple myeloma. Transl Cancer Res. 2023;12:3074–87. 38130312 10.21037/tcr-23-960 PMC10731335

[137] Thapa R, et al. New horizons in lung cancer management through ATR/CHK1 pathway modulation. Future Med Chem. 2023;15:1807–18. 37877252 10.4155/fmc-2023-0164

[138] Zhang L, et al. Cuproptosis combined with lncRNAs predicts the prognosis and immune microenvironment of breast cancer. Comput Math Methods Med. 2022;2022:5422698. 36213577 10.1155/2022/5422698 PMC9536992

[139] Cui L, et al. Radiosensitization by gold nanoparticles: will they ever make it to the clinic? Radiother Oncol. 2017;124:344–56. 28784439 10.1016/j.radonc.2017.07.007

[140] Bettigole SE, Glimcher LH. Endoplasmic reticulum stress in immunity. Annu Rev Immunol. 2015;33:107–38. 25493331 10.1146/annurev-immunol-032414-112116

[141] Wang H, Guo M, Wei H, Chen Y. Targeting p53 pathways: mechanisms, structures, and advances in therapy. Signal Transduct Target Ther. 2023. 10.1038/s41392-023-01347-1. 36859359 10.1038/s41392-023-01347-1 PMC9977964

[142] Kamenov K, Twomey C, Cabello M, Prina AM, Ayuso-Mateos JL. The efficacy of psychotherapy, pharmacotherapy and their combination on functioning and quality of life in depression: a meta-analysis. Psychol Med. 2017;47:414–25. 27780478 10.1017/S0033291716002774 PMC5244449

[143] Huang AC, Zappasodi R. A decade of checkpoint blockade immunotherapy in melanoma: understanding the molecular basis for immune sensitivity and resistance. Nat Immunol. 2022;23:660–70. 35241833 10.1038/s41590-022-01141-1 PMC9106900

[144] Brownmiller T, et al. Y chromosome lncRNA are involved in radiation response of male non-small cell lung cancer cells. Cancer Res. 2020;80:4046–57. 32616503 10.1158/0008-5472.CAN-19-4032 PMC7541653

[145] Bhan A, Soleimani M, Mandal SS. Long noncoding RNA and cancer: a new paradigm. Cancer Res. 2017;77:3965–81. 28701486 10.1158/0008-5472.CAN-16-2634 PMC8330958

[146] Bakhoum SF, et al. Numerical chromosomal instability mediates susceptibility to radiation treatment. Nat Commun. 2015. 10.1038/ncomms6990. 25606712 10.1038/ncomms6990 PMC4516720

[147] Du T, et al. Pyroptosis, metabolism, and tumor immune microenvironment. Clin Transl Med. 2021. 10.1002/ctm2.492. 34459122 10.1002/ctm2.492 PMC8329701

[148] Elhanani O, Ben-Uri R, Keren L. Spatial profiling technologies illuminate the tumor microenvironment. Cancer Cell. 2023;41:404–20. 36800999 10.1016/j.ccell.2023.01.010

[149] Jarosz-Biej M, Smolarczyk R, Cichoń T, Kułach N. Tumor microenvironment as a ‘game changer’ in cancer radiotherapy. Int J Mol Sci. 2019. 10.3390/ijms20133212. 31261963 10.3390/ijms20133212 PMC6650939

[150] Yang W, et al. Exosomes from young healthy human plasma promote functional recovery from intracerebral hemorrhage via counteracting ferroptotic injury. Bioact Mater. 2023;27:1–14. 37006825 10.1016/j.bioactmat.2023.03.007 PMC10060149

[151] D’Angelo E, Agostini M. Long non-coding RNA and extracellular matrix: the hidden players in cancer-stroma cross-talk. Noncoding RNA Res. 2018;3:174–7. 30533566 10.1016/j.ncrna.2018.08.002 PMC6260485

[152] Shi Y, et al. Cuproptosis-related lncRNAs predict prognosis and immune response of thyroid carcinoma. Front Genet. 2023. 10.3389/fgene.2023.1100909. 37470034 10.3389/fgene.2023.1100909 PMC10352785

[153] Gong H, et al. Identification of cuproptosis-related lncRNAs with the significance in prognosis and immunotherapy of oral squamous cell carcinoma. Comput Biol Med. 2024. 10.1016/j.compbiomed.2024.108198. 38417385 10.1016/j.compbiomed.2024.108198

[154] Hajjari M, Salavaty A. HOTAIR: an oncogenic long non-coding RNA in different cancers. Cancer Biol Med. 2015;12:1–9. 25859406 10.7497/j.issn.2095-3941.2015.0006 PMC4383848

[155] Sun C, et al. Tumor-associated nonmyelinating schwann cell-expressed PVT1 promotes pancreatic cancer kynurenine pathway and tumor immune exclusion. Sci Adv. 2023. 10.1126/sciadv.add6995. 36724291 10.1126/sciadv.add6995 PMC9891701

[156] Sun X, et al. Characterization of cuproptosis-related lncRNA landscape for predicting the prognosis and aiding immunotherapy in lung adenocarcinoma patients. Am J Cancer Res. 2023;13:778–801. 37034212 PMC10077038

[157] Han J, Luo J, Wang C, Kapilevich L, Zhang XA. Roles and mechanisms of copper homeostasis and cuproptosis in osteoarticular diseases. Biomed Pharmacother. 2024;174:116570. 38599063 10.1016/j.biopha.2024.116570

[158] Tung CH, et al. Α-catulin promotes cancer stemness by antagonizing WWP1-mediated KLF5 degradation in lung cancer. Theranostics. 2022;12:1173–86. 35154481 10.7150/thno.63627 PMC8771551

[159] Liu Y, et al. A novel EHD1/CD44/Hippo/SP1 positive feedback loop potentiates stemness and metastasis in lung adenocarcinoma. Clin Transl Med. 2022. 10.1002/ctm2.836. 35485206 10.1002/ctm2.836 PMC9786223

[160] Yin H, et al. Identification of active bronchioalveolar stem cells as the cell of origin in lung adenocarcinoma. Cancer Res. 2022;82:1025–37. 35045987 10.1158/0008-5472.CAN-21-2445

[161] Kopp F, Mendell JT. Functional classification and experimental dissection of long noncoding RNAs. Cell. 2018;172:393–407. 29373828 10.1016/j.cell.2018.01.011 PMC5978744

[162] Pan J, et al. LncRNA JPX/miR-33a-5p/Twist1 axis regulates tumorigenesis and metastasis of lung cancer by activating Wnt/β-catenin signaling. Mol Cancer. 2020. 10.1186/s12943-020-1133-9. 31941509 10.1186/s12943-020-1133-9 PMC6961326

[163] Schmitt AM, Chang HY. Long noncoding RNAs in cancer pathways. Cancer Cell. 2016;29:452–63. 27070700 10.1016/j.ccell.2016.03.010 PMC4831138

[164] Liu J, et al. Wnt/β-catenin signalling: function, biological mechanisms, and therapeutic opportunities. Signal Transduct Target Ther. 2022. 10.1038/s41392-021-00762-6. 34980884 10.1038/s41392-021-00762-6 PMC8724284

[165] Lin S, Zhen Y, Guan Y, Yi H. Roles of Wnt/β-catenin signaling pathway regulatory long non-coding RNAs in the pathogenesis of non-small cell lung cancer. Cancer Manag Res. 2020;12:4181–91. 32581590 10.2147/CMAR.S241519 PMC7280066

[166] Huang J, Xu Z, Yuan Z, Teh BM, Zhou C, Shen Y. Identification of a cuproptosis-related lncRNA signature to predict the prognosis and immune landscape of head and neck squamous cell carcinoma. Front Oncol. 2022;12:983956. 36568234 10.3389/fonc.2022.983956 PMC9780454

[167] Baba SK, et al. Long non-coding RNAs modulate tumor microenvironment to promote metastasis: novel avenue for therapeutic intervention. Front Cell Dev Biol. 2023. 10.3389/fcell.2023.1164301. 37384249 10.3389/fcell.2023.1164301 PMC10299194

[168] Riquelme I, et al. Long non-coding RNAs (lncRNAs) as regulators of the PI3K/AKT/mTOR pathway in gastric carcinoma. Int J Mol Sci. 2023. 10.3390/ijms24076294. 37047267 10.3390/ijms24076294 PMC10094576

[169] Sun L, Chen X, Li F, Liu S. Construction and significance of a breast cancer prognostic model based on cuproptosis-related genotyping and lncRNAs. J Formos Med Assoc. 2024. 10.1016/J.JFMA.2024.05.007. 38772805 10.1016/j.jfma.2024.05.007

[170] Wang K, et al. Long non-coding RNAs in ferroptosis and cuproptosis impact on prognosis and treatment in hepatocellular carcinoma. Clin Exp Med. 2024. 10.1007/s10238-024-01397-x. 38907744 10.1007/s10238-024-01397-x PMC11193701

[171] Chen W, Ruan M, Zou M, Liu F, Liu H. Clinical significance of non-coding RNA regulation of programmed cell death in hepatocellular carcinoma. Cancers (Basel). 2023. 10.3390/cancers15164187. 37627215 10.3390/cancers15164187 PMC10452865

[172] Zhang X, et al. Copper-mediated novel cell death pathway in tumor cells and implications for innovative cancer therapies. Biomed Pharmacother. 2023. 10.1016/j.biopha.2023.115730. 37864891 10.1016/j.biopha.2023.115730

[173] Baldari S, Rocco GD, Toietta G. Current biomedical use of copper chelation therapy. Int J Mol Sci. 2020. 10.3390/ijms21031069. 32041110 10.3390/ijms21031069 PMC7037088

[174] Talib WH, Alsayed AR, Barakat M, Abu-Taha MI, Mahmod AI. Targeting drug chemo-resistance in cancer using natural products. Biomedicines. 2021. 10.3390/biomedicines9101353. 34680470 10.3390/biomedicines9101353 PMC8533186

[175] Yang L, et al. Cuproptosis-related lncRNAs are biomarkers of prognosis and immune microenvironment in head and neck squamous cell carcinoma. Front Genet. 2022;13:947551. 35938003 10.3389/fgene.2022.947551 PMC9354258

[176] Cui G, et al. Comprehensive analysis of the prognostic signature and tumor microenvironment infiltration characteristics of cuproptosis-related lncRNAs for patients with colon adenocarcinoma. Front Oncol. 2022;12:1007918. 36212459 10.3389/fonc.2022.1007918 PMC9539748

[177] Ma C, et al. Prognosis and personalized treatment prediction in lung adenocarcinoma: an in silico and in vitro strategy adopting cuproptosis related lncRNA towards precision oncology. Front Pharmacol. 2023;14:1113808. 36874011 10.3389/fphar.2023.1113808 PMC9975170

[178] Gaur K, et al. Iron and copper intracellular chelation as an anticancer drug strategy. Inorganics. 2018. 10.3390/inorganics6040126. 33912613 10.3390/inorganics6040126 PMC8078164

[179] Wang Y, et al. Cuproptosis: a novel therapeutic target for overcoming cancer drug resistance. Drug Resist Updat. 2024. 10.1016/j.drup.2023.101018. 37979442 10.1016/j.drup.2023.101018

[180] Huang G, et al. Identification of cuproptosis-related long noncoding RNA signature for predicting prognosis and immunotherapy response in bladder cancer. Sci Rep. 2022;12:21386. 36496537 10.1038/s41598-022-25998-2 PMC9741610

[181] Ge H, et al. Targeting ASIC1a promotes neural progenitor cell migration and neurogenesis in ischemic stroke. Research. 2023. 10.34133/research.0105. 37275123 10.34133/research.0105 PMC10234266

[182] Leso V, et al. Exposure to antineoplastic drugs in occupational settings: a systematic review of biological monitoring data. Int J Environ Res Public Health. 2022. 10.3390/ijerph19063737. 35329423 10.3390/ijerph19063737 PMC8952240

[183] Pulumati A, Pulumati A, Dwarakanath BS, Verma A, Papineni RVL. Technological advancements in cancer diagnostics: improvements and limitations. Cancer Rep. 2023. 10.1002/cnr2.1764. 10.1002/cnr2.1764 PMC9940009 36607830

[184] Hueso M, et al. ncRNAs in therapeutics: challenges and limitations in nucleic acid-based drug delivery. Int J Mol Sci. 2021. 10.3390/ijms222111596. 34769025 10.3390/ijms222111596 PMC8584088

[185] Zhou H, Toan S. Pathological roles of mitochondrial oxidative stress and mitochondrial dynamics in cardiac microvascular ischemia/reperfusion injury. Biomolecules. 2020. 10.3390/biom10010085. 31948043 10.3390/biom10010085 PMC7023463

